# Research on Seafloor 3D Reconstruction Method Based on Sparse Measurement Points

**DOI:** 10.3390/s26020639

**Published:** 2026-01-18

**Authors:** Erliang Xiao, Lang Qin, Zhipeng Chi, Haiqing Gu, Yunsong Hua, Hui Yang, Ran Li

**Affiliations:** School of Optical-Electrical and Computer Engineering, University of Shanghai for Science and Technology, Shanghai 200093, China; xiaoerliang@usst.edu.cn (E.X.); 232260490@st.usst.edu.cn (L.Q.); lwxiaochi2019@163.com (Z.C.); 232210422@st.usst.edu.cn (H.G.); hyuns_yz@163.com (Y.H.); yanghui@usst.edu.cn (H.Y.)

**Keywords:** seafloor 3D reconstruction, sparse measurement points, fractal algorithm, Gaussian model

## Abstract

Seafloor 3D reconstruction is a core technology for seafloor topography and deformation monitoring. Due to the complexity of the deep-sea environment and the high requirements for measurement devices, long-term monitoring can only acquire low-resolution and limited seafloor topography data. This leads to difficulties for existing 3D reconstruction algorithms in handling details and accuracy, especially with complex variations in seafloor terrain, which poses higher demands on 3D reconstruction algorithms. This study proposes a “fractal–Gaussian process” hybrid model, leveraging the fractal self-similarity property to precisely capture complex local details of the seafloor terrain, combined with the Bayesian global optimization ability of the Gaussian process model, to achieve high-resolution modeling of seafloor 3D reconstruction. Finally, Perlin noise is introduced to enhance the naturalness and detail representation of the terrain. Experiments show that under sparse data conditions, the proposed method significantly outperforms traditional interpolation methods, with average errors reduced by 30–40% and an R^2^ value of 0.9836.

## 1. Introduction

In ocean exploration and resource extraction engineering, monitoring seafloor topography and deformation is of significant importance [1]. Especially in the complex deep-sea environment, conventional acoustic and optical methods struggle to provide long-term, low-cost monitoring. Seafloor data collection often relies on a limited number of sparse discrete monitoring points [2]. These sparse monitoring point data cannot directly describe the complex seafloor topography. Therefore, efficiently and accurately reconstructing 3D models from these data is a key issue in both seafloor topography research and engineering applications.

3D reconstruction technology is a computer technique used to restore the three-dimensional geometric structure and surface properties of objects from two-dimensional images, point cloud data, or other sensor information [3]. The key challenge of this technology is how to achieve high-precision surface reconstruction with limited observation data, which involves solving core problems such as data registration, noise filtering, and computational efficiency. Due to the high pressure and darkness of the deep-sea environment, the use of high-resolution optical equipment is limited, and sonar and other sensors typically only obtain sparse and discrete depth data. Therefore, the reconstruction of deep-sea seafloor morphology should focus on 3D reconstruction methods based on sparse measurement points [4]. Spatial interpolation is a technique used to estimate the attribute value of a target location based on the attribute values of neighboring points [5]. Its core principle is based on Tobler’s First Law of Geography [6], which states that everything is related to everything else, and the closer things are, the stronger their relationship [7]. Therefore, spatially adjacent points tend to have high attribute value similarity, allowing the attribute values of neighboring points to predict the target point’s attribute value [8].

Spatial interpolation methods can be divided into deterministic methods and probabilistic methods. Deterministic spatial interpolation methods typically assume that data follow a known pattern or empirical model in space, without considering randomness or errors in the data. These methods are suitable for relatively simple data situations or applications where high precision is not particularly required [9]. Examples include polynomial interpolation [10], linear interpolation [11,12], B-spline functions [13], etc. However, these methods are unable to model seafloor topography. The Inverse Distance Weighting (IDW) method [14], due to its small computational load and simple implementation, has become an important interpolation tool in many GIS software packages (GIS Box v2.0.9) [15,16]. However, the distance–decay relationship assumed by the IDW method is usually constant and isotropic across the entire study area, which may fail to precisely capture subtle terrain variations in complex topographies, resulting in the loss of edge details and impacting the overall terrain representation [17].

Probabilistic methods rely on spatial autocorrelation and consider both distance and direction to evaluate the importance of each sampling point for the interpolation result [18]. For instance, the Kriging interpolation algorithm, proposed by South African mining geographer Krige [19], is based on the variogram theory in geostatistics. It utilizes the spatial autocorrelation of data to make predictions [20] and has been widely applied in fields such as geostatistics and image processing [21]. The Radial Basis Function (RBF) interpolation method fits the data by defining a weight function similar to Kriging [22]. Random field models are another class of probabilistic interpolation methods [23], which treat spatial data as a type of random process and construct models based on the statistical characteristics of the data to perform spatial interpolation. Although these methods possess strong predictive capabilities when handling spatial data, their computational complexity, sensitivity to data quality, and dependency on parameter and model selection pose limitations in practical applications [24].

With the increasing attention on deep learning technology, some deep learning [25] and machine learning [26] interpolation algorithms have also started to be used in the field of 3D reconstruction. Fuzzy clustering-based interpolation algorithms [27], generative adversarial networks [28], and other methods have shown excellent performance in small-scale data filling tasks, but their accuracy and efficiency significantly decrease when dealing with large-scale data filling. Gaussian Process Regression (GPR) [29,30,31], as a Bayesian non-parametric regression technique, can generate terrain models of arbitrary resolution through its probabilistic inference capabilities [32], focusing on how to efficiently and accurately represent and process large-scale terrain data [33]. However, when the data volume is small or the relationships between data features are poor, this method cannot yield better results. Therefore, combining these spatial interpolation algorithms with deep learning methods is a highly promising technical solution. A spatial interpolation method based on a clustering adaptive inverse distance weighting algorithm and linear regression [34] solves the drawbacks of traditional inverse distance weighting methods in spatial interpolation when data distribution is uneven or there are outliers. However, parameters such as the number of clusters and the distance exponent for weighting significantly affect the interpolation results. The fractal interpolation algorithm differs from these traditional methods [35], as it interpolates and generates new data points using fractal functions. Its core idea is to iteratively construct curves or surfaces with self-similarity in space [36], which appear to have fractal characteristics and can approximate the target spatial data points, making it suitable for processing spatial data with self-similarity or complex structures [37].

In this paper, we propose a method that combines fractal interpolation system (fis) with GPR. Based on the self-similar characteristics of the seabed 3D model, the fractal interpolation function algorithm is used to capture local terrain information, and then, using the non-parametric GPR for global regression, the global information is utilized to smooth and fit the data, complementing the advantages of both methods. Finally, Perlin noise is added to optimize the simulated terrain [38]. The remainder of this paper is organized as follows. Section 2 introduces the experimental system and research methods. Section 3 details the experimental and analysis. Section 4 presents the discussion. Finally, Section 5 provides the conclusion.

## 2. Experimental Method

### 2.1. Design of the Terrain Deformation 3D Reconstruction Model

**Figure 1:**
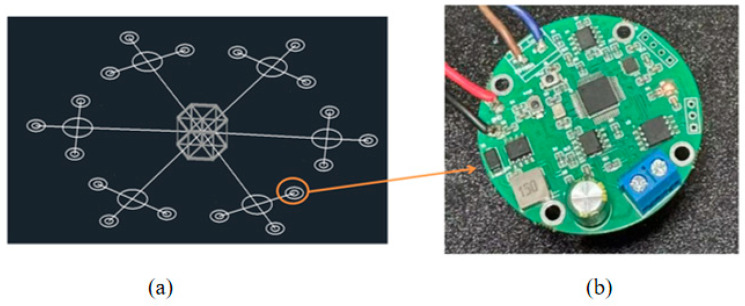
Seabed Topography Detection and Deformation Monitoring System Design: (**a**) System Diagram—Layout Method; (**b**) Data Collection—Inertial Navigation Principle.

This project was conducted under the funding of the “14th Five-Year Plan” major project of the Qingdao National Laboratory for Marine Science and Technology, University of Shanghai for Science and Technology. A long-term seafloor terrain monitoring system has been designed and developed based on inertial measurement technology. The system consists of inertial measurement nodes and rigid connecting rods, forming a star-shaped structure, as shown in Figure 1a. The node shown in Figure 1b is an IMU (Inertial Measurement Unit) node, equipped with a three-axis gyroscope that records the angular velocity changes in an object around the X, Y, and Z axes, reflecting the object’s rotational state and angular variations. Each IMU can comprehensively monitor the state of surrounding moving objects. By working together, multiple IMUs can accurately track movements within a larger monitoring area. Each secondary monitoring node is equipped with three IMUs, allowing them to collect motion data from different angles and directions, thus ensuring the comprehensiveness and accuracy of the collected data.

The bending phenomenon refers to the rotation of the sensor around the Y and Z axes, represented as pitch and yaw angles, respectively. Torsion is represented by rotation around the X axis, the roll angle. These three are collectively referred to as Euler angles. The accelerometer in the sensor can provide reference information on gravitational acceleration when the system is stationary or moving at a constant speed. Based on the gravitational components along each axis of the three-axis accelerometer in stationary or constant speed states, the current tilt angle of the accelerometer can be calculated. By measuring these two angles, the specific orientation of the sensor in three-dimensional space can be obtained. Using the attitude data from these sensor nodes, the spatial position of the seabed surface after deformation can be further determined, and a three-dimensional seabed terrain deformation vector model can be established.

**Figure 2:**
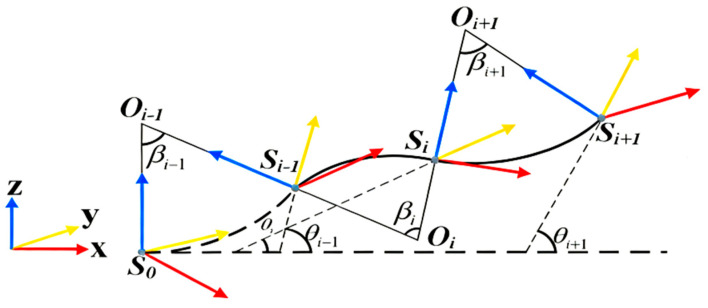
Principle of Inertial Navigation Topography Measurement.

As shown in Figure 2, based on the sensor’s bending and twisting angles, the pitch angle of the sensor node is calculated using the three-axis accelerometer. By combining the initial positions of the sensor array, the spatial position of each sensor can be determined, thus obtaining the entire seabed terrain in the monitoring area. For the sensor array, terrain deformation such as settlement or uplift can be considered as a two-dimensional change in the vertical direction. To simplify the calculation, the deformation between sensors is approximated using an arc. This approximation method can effectively describe the deformation of the sensor nodes and enable three-dimensional reconstruction.

The central angle of the arc Si + 1 can be represented by Formula (1):

(1) $$\beta_{i}=\theta_{i+1}-\theta_{i}$$

where θi and θi + 1 are the pitch angles of the sensor Si and sensor Si + 1, respectively. The radius of the arc is (l is the distance between adjacent sensors), and the coordinates of the center of the arc Oi in the coordinate system Oi-Xi-Yi-Zi (with sensor Si as the origin) and the coordinates of the endpoint in the coordinate system Oi-Xi-Yi-Zi can be represented by Formula (2).

(2) $$S_{i+1}^{i}={\left[dx,0,dz\right]}^{T}=\left\{\begin{array}{l} \left[r_{i}\sin\beta_{i},0,r_{i}-r_{i}\cos\beta_{i}{]}^{T},(\beta \neq 0\right)\\ \left[l,0,0{]}^{T},(\beta =0\right) \end{array}\right.$$

The endpoint in the reference coordinate system O-X-Y-Z can be represented by Formula (3):

(3) $$\left[\begin{array}{c} S_{i=1}^{0}\\ 1 \end{array}\right]=H_{i}^{0}\left[\begin{array}{c} S_{i+1}^{i}\\ 1 \end{array}\right]=\left[\begin{array}{cc} C_{i}^{0} & S_{i}^{0}\\ O_{3}^{T} & 1 \end{array}\right]\left[\begin{array}{c} S_{i+1}^{i}\\ 1 \end{array}\right]$$

where (R) is the rotation matrix from the reference coordinate system O-X-Y-Z to the coordinate system Oi-Xi-Yi-Zi, which can be represented by Formula (4):

(4) $$C_{i}^{0}=C_{i-1}^{0}C_{i}^{i-1}$$

In the new coordinate system, the position of the next sensor can be calculated, and by recursively applying this process, the positions of all the sensors in the original coordinate system can be obtained.

### 2.2. Fractal Algorithm

Fractal graphics exhibit self-similarity or self-affinity, where the local part is a smaller replica of the whole. This replication can be generalized as an affine transformation. The Fractal Iterated Function System (IFS) is based on this idea. It typically assumes that both the overall shape and its local parts possess a self-similar structure in terms of affine transformations. By defining the overall shape and selecting a number of affine transformations, the overall form is transformed into local parts. This process is iteratively applied until a satisfactory graphic is obtained.

**Figure 3:**
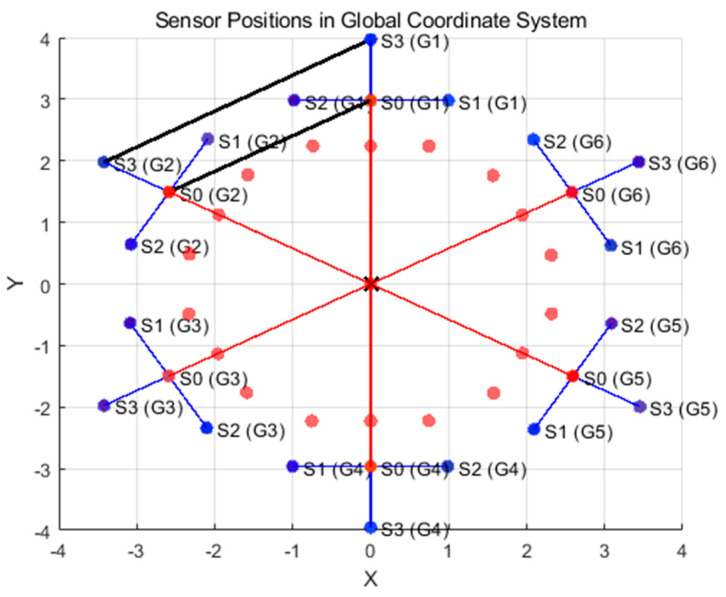
Region Partitioning Method.

For some regular shapes, the corresponding IFS affine parameters can be determined using their obvious geometric relationships. For shapes with irregular boundaries, a block-based approach can be used. Figure 3 demonstrates the partitioning of a polygonal region. This region is reconstructed from spatial data collected by six groups of sensors through the arc model.

In this case, the connecting rods of two adjacent sensor groups are treated as two edges of a triangle, with the central origin as one vertex of the triangle. The two adjacent sensors, S0 and S3, are connected to form the third edge of the triangle. This way, two triangles can be obtained.

The initial triangle region consists of the origin O, S4(G1), and S4(G2). The positions of these points on the coordinate plane are denoted as: (x_n1_, y_n1_, z_n1_), (x_n2_, y_n2_, z_n2_) and (x_n3_, y_n3_, z_n3_).

After mapping, the new triangle is formed by the points O, S0(G1), and S0(G2), with their corresponding positions on the coordinate plane marked as: (x_1_, y_1_, z_1_), (x_2_, y_2_, z_2_) and (x_3_, y_3_, z_3_).

The region to be mapped is the one where interpolation is required. To calculate the mapping, the method is to solve a system of linear equations by matching the corresponding three vertices of the two triangles (before and after mapping). The unknowns are obtained by solving these equations. In summary, the fractal algorithm applies affine transformations iteratively, using geometric relationships and linear interpolation between corresponding points of triangles to map the irregular regions. This method is particularly effective for handling sensor data and creating a smooth transformation of spatial locations in a defined space.

Its affine transformation can be represented by Equation (5).

(5) $$[\begin{array}{ccc} x_{1} & x_{2} & x_{3}\\ y_{1} & y_{2} & y_{3}\\ z_{1} & z_{2} & z_{3} \end{array}]=M[\begin{array}{ccc} x_{n1} & x_{n2} & x_{n3}\\ y_{n1} & y_{n2} & y_{n3}\\ z_{n1} & z_{n2} & z_{n3} \end{array}]+T$$

Here, M is a 3 × 3 matrix representing the linear transformation, and T is a 3 × 1 vector representing the translation. By solving the above linear system of equations, the affine transformation matrix M and the translation vector T can be obtained. Then, by applying the affine transformation to all the points in the original triangular region (the blue points), they are mapped to the smaller triangular region, resulting in new red points. This process can be repeated for the entire region by first dividing it into smaller regions and then applying the transformation to obtain a new set of points.

### 2.3. Gaussian Process Model

GPR is a non-parametric regression method. In machine learning, GPR is used to model continuous function relationships. When making predictions, GPR calculates the posterior distribution of the target function given the input, which is also a Gaussian distribution. The mean of this posterior distribution is the predicted value. Since GPR is based on Bayesian inference, it can provide uncertainty estimates for the predictions, where the variance represents the uncertainty in the predictions. This is highly useful in many application scenarios.

For any arbitrary set X, a GPR defined on X is a collection of random variables such that for any set of inputs, the joint distribution is a multivariate Gaussian distribution. Therefore, a GPR is uniquely determined by the mean function and the covariance function K (also called the kernel function), commonly represented by Equation (6).

(6) $$f\left(x\right)\sim GP\left(\mu \left(x\right),k\left(x,x^{\prime }\right)\right)$$

The function value at a new point is predicted. According to the properties of the Gaussian distribution, the joint distribution of the training points and the prediction points remains a Gaussian distribution, as shown in Equation (7).

(7) $$\left[\begin{array}{c} y\\ f^{\prime } \end{array}\right]\sim N\left(\left[\begin{array}{c} \mu \left(x\right)\\ \mu \left(x^{\prime }\right) \end{array}\right],\left[\begin{array}{cc} k\left(x,x\right)+\sigma_{n}^{2}I & k{\left(x^{\prime },x\right)}^{T}\\ k\left(x^{\prime },x\right) & k\left(x^{\prime },x^{\prime }\right) \end{array}\right]\right)$$

where x represents the training data, x′ represents the test data, and k denotes the covariance matrix.

Finally, by using the conditional distribution property of the Gaussian distribution, the conditional probability distribution of the predicted value can be obtained, as shown in Equation (8).

(8) $$p\left(f^{\prime }|X,y,x^{\prime }\right)=N\left(\hat{\mu },\hat{\sigma }\right)$$

In practical applications, it is common to set the mean function to 0. Therefore, the predicted mean function and the predicted covariance function can be more concisely expressed as Equations (9) and (10):

(9) $$\hat{\mu }=k\left(x^{\prime },x\right){\left(k\left(x,x\right)+\sigma_{n}^{2}I\right)}^{-1}y$$

(10) $$\hat{\Sigma }=k\left(x^{\prime },x^{\prime }\right)-k\left(x^{\prime },x\right){\left(k\left(x,x\right)+\sigma_{n}^{2}I\right)}^{-1}k{\left(x^{\prime },x\right)}^{T}$$

By observing the above equation, we can identify some properties: First, for the mean, the test dataset contains m points, so it should be m × 1, while the corresponding right-hand side, denoted by y, should be n × 1. The other parts on the right-hand side, excluding y, should be m × n. Therefore, the predicted mean is a linear combination of the observed points y. Now, looking at the covariance, the first part on the right-hand side represents the prior covariance, and the subtracted part reflects the reduction in the uncertainty of the function distribution after observing the data. If the second term is very close to 0, it indicates that the uncertainty after observing the data remains almost unchanged. On the other hand, if the second term is very large, it means the uncertainty has been significantly reduced.

## 3. Simulation of Seafloor for Algorithm Validation

### 3.1. Comparison of Fractal Algorithm and the Method of Dividing the Rod into Equal Segments

Since there are no measurement points in the middle section, and considering the relationship between the mechanical structure of the rod and the terrain, the simulated terrain surface should be positioned below the rod to make the simulation results more reasonable. Therefore, it is necessary to know the location of the rod.

(1) Dividing the rod into equal segments at both ends and calculating the coordinates of the intermediate points

The rod has two endpoints, (p1) and (p2), with coordinates ((x1, y1, z1)) and ((x2, y2, z2)), respectively. Suppose we want to divide the rod into (N) equal segments. The distance between each segment is the distance from (p1) to (p2) divided by (N), meaning each segment has a length of (L/N). The index (i = (1, 2, ~, N)) corresponds to a specific position along the rod, from (p1) to (p2).

(11) $$L=\sqrt{{\left(x_{2}-x_{1}\right)}^{2}+{\left(y_{2}-y_{1}\right)}^{2}+{\left(z_{2}-z_{1}\right)}^{2}}$$

where (L) is the total length of the rod.

The coordinates of each division point can be calculated using the following Formula (12):

(12) pi=x1+i⋅x2−x1N,y1+i⋅y2−y1N,z1+i⋅z2−z1N

(2) The fractal interpolation method can simulate the transitional part between the rod and the surrounding terrain, especially in cases where the terrain changes significantly or is complex. Based on the position and shape of the rod, combined with the features of the terrain, and using the existing points, such as the endpoints of the rod and nearby terrain points, the fractal interpolation algorithm is applied to generate additional points, expanding the detailed position between the rod and the surrounding area.

The analysis of the four evaluation indicators comparing the fractal interpolation method and the equal division point coordinate method shows the following:

1. Mean Error: Fractal interpolation method: 0.0866; Equal division point coordinate method: 0.0974. The fractal interpolation method has a smaller mean error, indicating a more precise fit to the data overall.
2. Mean Squared Error: Fractal interpolation method: 0.0130; Equal division point coordinate method: 0.0225. The mean squared error of the fractal interpolation method is significantly lower, suggesting that it not only has a smaller error magnitude but also smaller squared errors, showing a better fitting performance.
3. Coefficient of Determination: Fractal interpolation method: 0.9558; Equal division point coordinate method: 0.9275. This indicates that the fractal interpolation method can better capture the variation trends of the data and has a higher model fitting degree.
4. Cross-validation Error: Fractal interpolation method: 0.01831; Equal division point coordinate method: 0.05444. The lower cross-validation error indicates stronger generalization ability, meaning the model can adapt to more diverse datasets. The fractal interpolation method has a significantly lower cross-validation error, showing better stability across different datasets.

**Figure 4:**
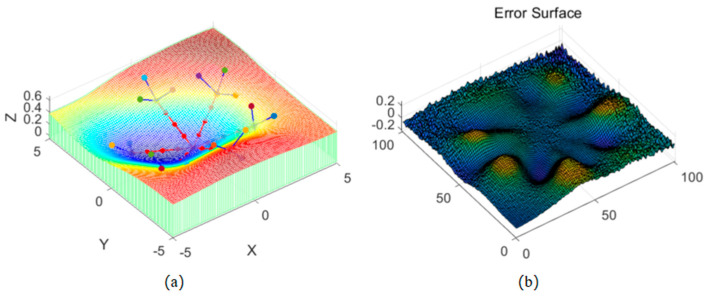
Method of Simulating Terrain by Dividing the Rod into Equal Segments: (**a**) known data points, divide (red) and Simulated terrain surface, (**b**) error surface.

**Figure 5:**
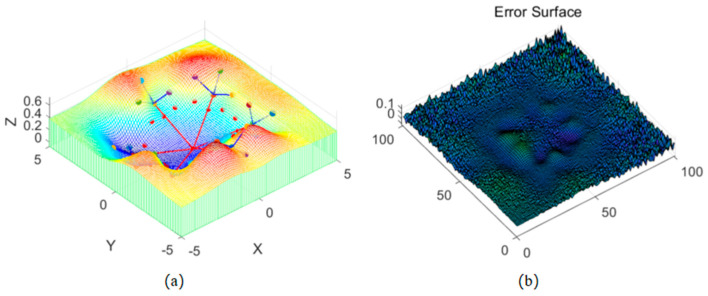
Terrain Simulation Using the Fractal Method: (**a**) known data points, Fractal Method calculated point (red) and Simulated terrain surface, (**b**) error surface.

As shown in Figure 4 and Figure 5, the advantages of the fractal interpolation method over the equal division point coordinate method mainly lie in its ability to precisely simulate the relationship between the rod and the complex terrain. The equal division point coordinate method simply distributes the rod’s position evenly, completely ignoring the influence of the surrounding terrain and failing to provide a smooth transition based on terrain changes. It assumes that the terrain is flat or uniform, overlooking terrain details, and cannot effectively reflect the characteristics of complex terrains. Therefore, the equal division point coordinate method cannot simulate deformations or special environmental interactions that the rod may encounter in complex terrain.

Points generated through the fractal algorithm capture terrain details, such as changes in slope, terrain undulations, and irregularities, which allows not only for determining the rod’s position on the terrain but also for simulating the natural transition between the rod and the surrounding terrain. This avoids abrupt or unrealistic bends and provides smooth, continuous simulation results. It reflects the real interaction between the rod and the terrain in simulations, effectively modeling complex natural terrains and surface changes.

### 3.2. Simulation Experiments of Different Terrain

In order to compare and analyze the interpolation accuracy and model fitting performance of common interpolation algorithms, such as the inverse distance weighting algorithm, Kriging interpolation algorithm, and GPR, in different terrains.

Different terrain types have their own unique characteristics. A concave terrain has a central area lower than the surrounding regions, resembling a basin, and can be simulated using a sine wave function with added noise. A convex terrain has a central area higher than the surrounding regions, resembling a hill, and can be simulated using a Gaussian distribution with added noise. An inclined terrain presents a gentle, unidirectional slope, similar to a ramp, and is typically modeled using a linear equation with slight noise disturbances. An irregular terrain exhibits significant surface undulations, containing various random fluctuations.

The following interpolation methods and their combinations will be compared: IDW assigns weights to surrounding known points based on the inverse of distance and predicts the elevation values of unknown points through weighted averaging. Kriging takes into account the spatial correlation between points, using a variogram to model the spatial dependence of the data, making it suitable for terrain data with spatial autocorrelation. GPR uses Bayesian inference to perform interpolation on unknown points through a GPR and provides uncertainty estimates.

We will adopt the authoritative marine elevation dataset provided by GEBCO (General Bathymetric Chart of the Oceans) (data access link: https://download.gebco.net/ (26 December 2025)), with the selected version being GEBCO 2025. This dataset integrates global multi-source marine bathymetric data (including multibeam sonar data, etc.) and can objectively reflect the topographic and geomorphic characteristics of the real seabed. The specific verification area will be the latitude and longitude range [n34.8431_s34.2444_w123.0634_e123.7335], and the data format will be selected from supported formats such as 2D netCDF, GeoTIFF, and Esri ASCII according to experimental needs.

#### 3.2.1. Setup and Process of Supplementary Verification Experiments

The setup and process will strictly follow the core scheme in the original paper to ensure the consistency of variable control and the comparability of results. Specifically, it includes keeping the parameter settings of the fractal model and Gaussian regression model unchanged, and adopting the same data preprocessing flow, model training steps, and accuracy evaluation indicators as those used in the synthetic terrain verification. By applying the models to the above-mentioned real seabed terrain data, the fitting effect and prediction accuracy of the models under real complex terrain conditions will be compared and analyzed.

Two classical fractal feature quantification methods were adopted for validation: (1) Based on the power-law relationship between the box size and the number of non-empty boxes, the fractal dimension was calculated via the box-counting method; (2) A variogram of terrain elevation was constructed to verify its power-law scaling characteristics across different lag distances, and the significance of the power-law relationship was determined by the goodness of fit.

#### 3.2.2. Experimental Results

Box-counting method: The goodness of fit reached R^2^ = 0.968 (>0.9, meeting the significance criterion), and the calculated fractal dimension was 2.256, which falls within the theoretical range of 2.1~2.9 for natural terrains. Variogram analysis: The goodness of fit was R^2^ = 0.972, and the slope of the power-law fitting was 1.389, which is consistent with the theoretical range of 1~2 for fractal terrains. The derived Hurst exponent was H = 0.6945, indicating strong persistence of the terrain.

The validation results collectively demonstrate that the seabed terrain in the study area exhibits significant and stable fractal self-similarity, and all core quantitative indicators conform to the theoretical requirements for fractal terrains. This finding provides solid quantitative evidence for the application of the fractal model in the present study.

Leveraging the FIS, we generate a supplementary set of points from the raw topographic dataset. Experimental results verify that these FIS-derived points exhibit minimal mean error. Therefore, we fuse the original points with the FIS-calculated points as the input of the GPR model, thereby realizing the simulation of a full-coverage terrain surface.

**Figure 6:**
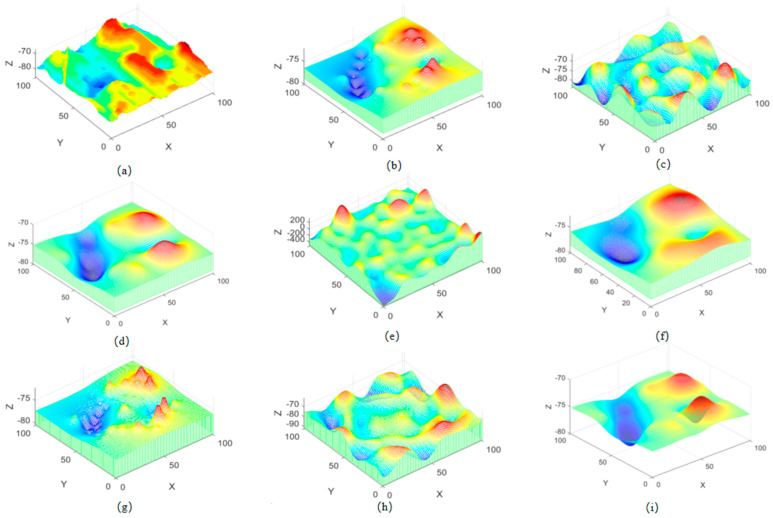
Terrain results simulated by single algorithms and combined algorithms: (**a**) Original image, (**b**) IDW, (**c**) Kriging, (**d**) GPR, (**e**) IDW + Kriging, (**f**) IDW + GPR, (**g**) fis + IDW, (**h**) fis + Kriging, (**i**) fis + GPR.

Figure 6a shows the real terrain, while the other subfigures display the terrain simulation results obtained by the inverse distance weighting (IDW) method, Kriging interpolation method, Gaussian process regression (GPR) method, and the combined algorithm. As can be seen from Figure 6b–d, constrained by the limited data volume, the single algorithms fail to accurately reproduce the characteristics of the real terrain. In contrast, after the introduction of the fractal algorithm, the simulated terrain maps in Figure 6g–i exhibit a significantly higher degree of consistency with the real terrain.

**Figure 7:**
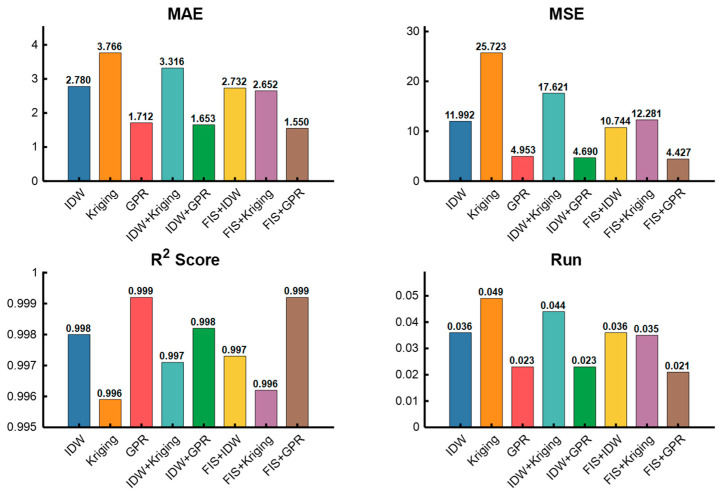
Comparison of performance indicators.

In the performance analysis of this set of terrain experiment simulations, the performance of different interpolation methods and models is compared from four perspectives: MAE, MSE, R^2^ Score, and RE, As shown in Figure 7:

Mean Absolute Error (MAE) Minimum value: fis + GPR (1.5499) Higher value: Kriging (3.7662). The lowest MAE is achieved by the fis + GPR method, indicating that the prediction results of this method are closest to the actual terrain values on average. IDW (2.7805) and GPR (1.7121) also yield relatively low MAE, showing good performance; in contrast, Kriging’s MAE is significantly higher than that of other methods, performing the worst in terms of average error.

Mean Squared Error (MSE) Minimum value: fis + GPR (4.4270) Higher value: Kriging (25.7228) MSE reflects the dispersion and stability of model prediction errors. The smallest MSE of fis + GPR indicates that its prediction errors have minimal fluctuations and optimal stability. IDW (11.9919) and GPR (4.9529) also fall into the low MSE range, demonstrating excellent performance; whereas Kriging’s MSE is much higher than other methods, resulting in the worst stability of prediction accuracy.

Coefficient of Determination (R^2^ Score) Maximum value: fis + GPR (0.9992) Minimum value: Kriging (0.9959). The R^2^ of fis + GPR is close to 1, indicating that this method achieves the best fitting effect on terrain data and can highly restore the distribution law of actual terrain. GPR (0.9992) achieves the same fitting effect as fis + GPR, also performing excellently; while Kriging’s R^2^ is the lowest among all methods, with relatively weak data fitting capability.

Relative Error (RE) Minimum value: fis + GPR (0.0206) Higher value: Kriging (0.0488). The smallest RE of fis + GPR indicates that it has the lowest relative deviation between prediction results and actual terrain values, achieving the optimal precision. GPR (0.0227) and IDW + GPR (0.0217) also maintain low RE levels; while Kriging’s RE is significantly higher, with the largest relative deviation.

Comprehensive Analysis of Overall Terrain Simulation Performance:

fis + GPR is the optimal method: It achieves the lowest values in three error indicators (MAE, MSE, RE) and an R^2^ score equal to GPR (close to 1). It not only ensures the “average proximity” between prediction results and actual terrain but also balances “error stability” and “data fitting degree”, making it the most suitable model for terrain interpolation scenarios in this experiment.

GPR-based methods (including combinations/fusions) perform outstandingly: Single GPR, IDW + GPR, and fis + GPR all outperform IDW, Kriging, and their combinations/fusions by a large margin in all indicators. This indicates that the inherent characteristics of the GPR model are more compatible with the distribution law of terrain data, and its advantages can be further amplified by combining with IDW and FIS.

Kriging has the poorest adaptability: Kriging ranks last in all indicators, indicating that the interpolation logic of this method has a low matching degree with the terrain data characteristics in this experiment, making it less suitable for simulating such terrain scenarios.

**Figure 8:**
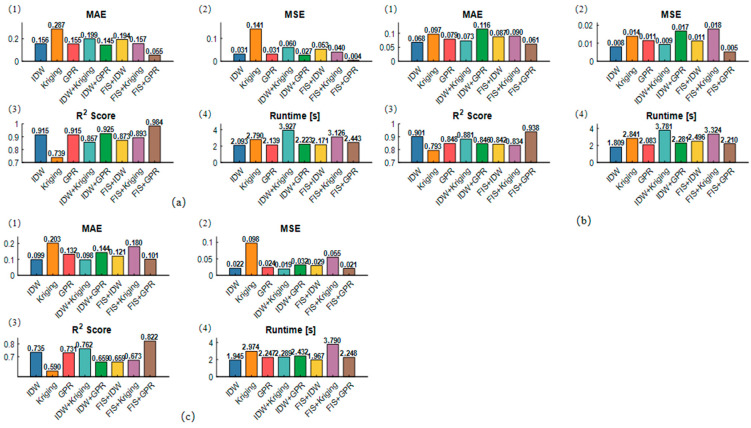
Comparison of performance indicators: (**a**) Comparison of performance indicators for different methods in concave terrain, (**b**) comparison of indicators for different methods in concave terrain, (**c**) comparison of indicators for different methods in sloping terrain.

Figure 8a,b overall compare the evaluation indicators for concave, convex, and sloping data, exploring the differences in performance under different terrain conditions. In the simulations for concave and convex terrains, the performance indicators of all methods are quite similar because concave and convex terrains have certain symmetrical characteristics in space. These can be observed as relative symmetry up and down or left and right from a central point or axis. In sloping terrain, the errors of all methods are higher, while in concave and convex terrain, the performance of the methods is significantly better.

Figure 8c shows that in sloping terrain, data points are often concentrated in areas with small elevation changes, while in areas with steeper slopes, the distribution of data points may be sparse. This uneven distribution affects the model’s training and prediction performance, leading to difficulties in interpolation and fitting. For example, IDW usually performs poorly in sloping terrain because it relies on the inverse distance weight, and in steep areas where elevation changes drastically, the distance differences become too large, causing inaccurate interpolation. Although Kriging is more complex, it relies on spatial autocorrelation relationships. In sloping terrain, the steep changes in slope and elevation make these spatial autocorrelation relationships unstable, causing Kriging to fail in effectively capturing terrain changes and resulting in large prediction errors. Furthermore, in sloping terrain, local changes (such as small slope variations or minor terrain fluctuations) have a significant impact on the overall structure, but many common models struggle to capture these details, especially in large-scale sloping terrains where models tend to focus more on overall trends and overlook local variations, leading to poor prediction performance.

The experimental analysis of the impact of fractal interpolation functions on terrain simulation. Without using the fractal algorithm, the outward mapping within the 50 × 50 range had an average error of 0.0784, a mean square error of 0.0157, and an R^2^ value of 0.7981. In comparison, after using the fractal algorithm, the average error within the 60 × 60 range significantly decreased to 0.0529, the mean square error reduced to 0.0058, and the R^2^ value increased to 0.9223.

By extending the measurement range with fractal interpolation, the overall terrain data can be better fitted, reducing overall error. The deviation of the predicted results from the actual values in terms of squared error shows a significant reduction, and the accuracy over larger areas is improved. After using the fractal algorithm, the R^2^ value increased significantly, improving the model’s fit and thus providing more accurate terrain simulation. This demonstrates that the method can effectively capture the self-similarity characteristics of the terrain, optimize the interpolation results for unmeasured areas, and improve the prediction of large-scale terrain. Therefore, using the fractal algorithm has clear advantages in terms of improving accuracy and expanding the simulation range.

**Figure 9:**
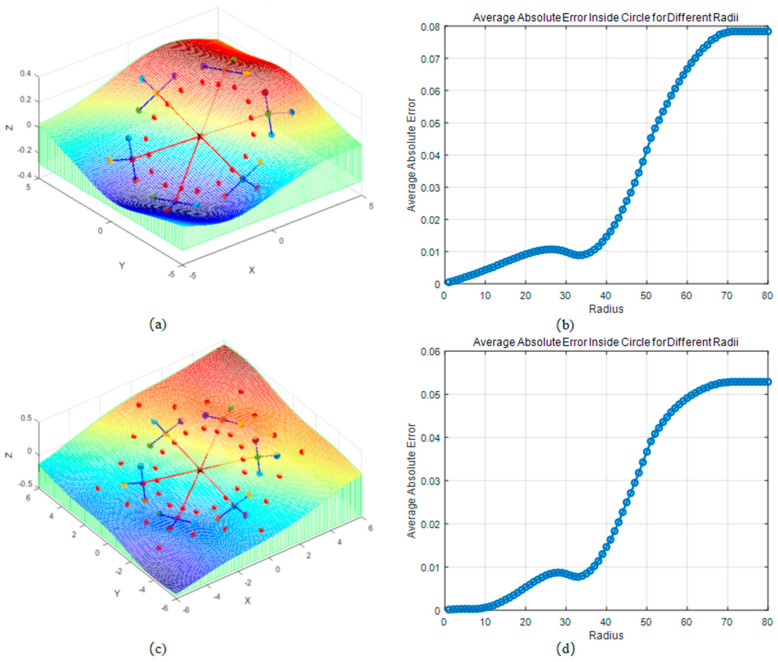
Without Fractal Algorithm for Outward Mapping and Fractal Algorithm for Outward Mapping: (**a**) points simulated by the fractal algorithm inward and Simulated terrain; (**b**) relationship between the radius of the simulation range and the average absolute error; (**c**) points simulated by the fractal algorithm inward and outward and Simulated terrain; (**d**) relationship between the radius of the simulation range and the average absolute error.

Figure 9b shows that the simulated terrain range is around 0 to 30, with relatively small average absolute errors. As the simulated terrain increases, there is a gradual rise in errors. When the simulated terrain range is between 30 and 40, due to the concentration of measured data in this range, the average absolute error shows a slight decreasing trend. Finally, as the simulation range exceeds 40, the average absolute error rises significantly, as the simulated terrain goes beyond the detectable range of the sensor.

Figure 9c shows the results with fractal interpolation for outward mapping (represented by blue dots). This method can effectively extend points beyond the measurement range, thus increasing the simulated terrain range. The overall trend is similar to Figure 9a,b, but with slight variations in the error values in certain areas. Figure 9d shows that in the range of 0 to 30, the overall error is slightly smaller, especially outside the measurement range. As the simulated terrain becomes larger, the error decreases. The fractal algorithm extends the simulation range by 20%. Fractal interpolation captures the complex variations in terrain through its self-similarity properties, especially for height variations and terrain structures commonly found in nature. It extends the spatial features of existing measurement points into unmeasured regions. This way, the model not only performs accurate interpolation within known areas but also generates reasonable terrain predictions outside the measurement range, simulating a broader range of terrain features. However, the applicability of this conclusion has a clear boundary, and its core limitation stems from the fundamental premise of the fractal algorithm: the universal assumption of terrain self-similarity does not hold in complex geological structures. When the terrain contains abrupt geological features that lack self-similarity, the prediction logic of the fractal algorithm will fail. The enhancement effect of the fractal algorithm on the interpolation model is scenario-dependent; it is suitable for scenarios where terrain self-similarity is stable and free from disturbances caused by abrupt geological structures. In areas with non-self-similar geological features such as fault lines and rock outcrops, the prediction accuracy of the algorithm will decrease, and it may even yield misleading results.

### 3.3. Optimizing the Generation of Seafloor Morphology Simulation

Existing terrain generation methods lack natural undulations and details, often producing abrupt or artificial traces, which makes them appear unrealistic and lacking in diversity. By incorporating Perlin noise for terrain optimization, it is possible to generate smooth and continuous terrain variations. Its pseudo-random characteristics and controllable parameters allow for fine-tuning of terrain features, while also providing high computational efficiency. This makes it suitable for large-scale real-time generation and supports multi-level detail optimization, thereby improving performance and visual effects.

**Figure 10:**
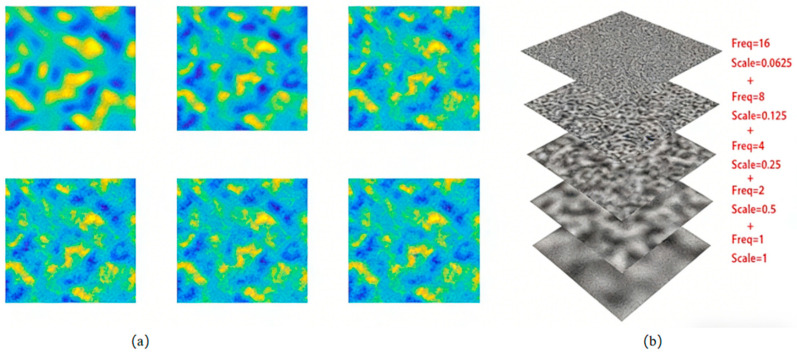
Perlin Noise Diagrams: (**a**) Noise Spectrum with Different Frequencies and Amplitudes; (**b**) Superposition of Noise with Different Frequencies and Amplitudes.

Perlin noise has amplitude and frequency similar to a sine wave. Although it does not exhibit the periodic regularity of a sine wave, the amplitude and frequency still show reasonable deviations within a certain range. Figure 10a shows 2D Perlin noise at frequencies of 1, 2, 4, 8, and 16:

Freq = 16, Scale = 0.0625: Represents high-frequency details with subtle textures.

Freq = 8, Scale = 0.125: Slightly lower frequency with reduced texture details.

Freq = 4, Scale = 0.25: Medium frequency, with textures starting to smooth out.

Freq = 2, Scale = 0.5: Low-frequency texture with fewer details.

Freq = 1, Scale = 1: The base layer, showing a very smooth surface with no noticeable texture.

The higher the frequency, the more refined the Perlin noise becomes, but the overall shape does not change drastically. This reflects natural phenomena. A common example is the contour of a mountain, which contains significant height variations (peaks), moderate variations (hills), small variations (gravel), and minute variations (stones). Therefore, low frequency and high amplitude are used to simulate large-scale changes, while high frequency and low amplitude are used to simulate small-scale changes.

As shown in Figure 10b, by superimposing Perlin noise with different frequencies, a more random waveform is obtained. It can be observed that the more frequencies are superimposed, the more detailed the noise becomes. The superimposed details maintain a macro-level similarity to the original waveform, which is a key feature of fractals.

**Figure 11:**
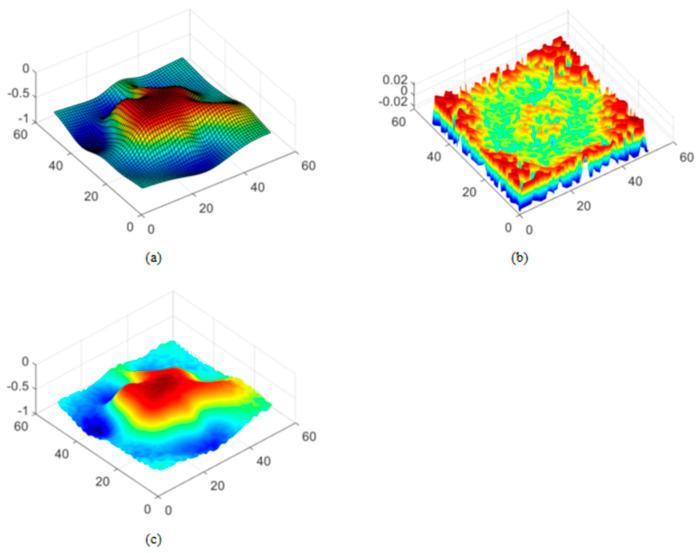
Perlin Noise Terrain Optimization: (**a**) Terrain Map Simulated Using GPR Model; (**b**) Generated Perlin Noise Map; (**c**) Terrain Map with Perlin Noise Added.

Experimental Results. As shown in Figure 11a, a known 3D coordinate dataset is used as training samples to construct a probabilistic model through GPR, outputting the global predicted mean and variance to estimate uncertainty. The variance values quantify the confidence of the model: low-variance regions indicate sufficient data or smooth terrain, while high-variance regions reflect missing data or significant terrain changes. The mean predicted by the GPR is used as the baseline elevation for the terrain, and Perlin noise is added as a layer of detail on top of the terrain generated by the GPR. Figure 11b shows a continuous, pseudo-random noise function, where frequency and amplitude adjustments introduce local variations and complexity into the terrain. The variance prediction is used as a weighting factor to control the amplitude of the Perlin noise. Normalization is applied to map the variance values proportionally to a reasonable amplitude range to avoid mismatched magnitudes. By leveraging the multi-frequency superposition characteristic of Perlin noise, more details are generated, ensuring a smooth transition with the macro terrain trends. Finally, Figure 11c shows that in dense observation areas, the high-confidence prediction from the GPR dominates the terrain shape, with the noise contribution being minimal. In sparse data areas, noise injection based on the variance generates reasonable details that align with the physical principles of the terrain.

## 4. Discussion

GPR has significant advantages over Kriging interpolation and inverse distance weighting methods. One of its core advantages is the ability to provide both predicted values and the uncertainty of the predictions (variance), which effectively analyzes the credibility of the predictions, especially in data-sparse areas. GPR optimizes the hyperparameters of the kernel function and comprehensively considers global data characteristics, achieving optimal overall fitting. Although Kriging also performs global fitting, its optimization typically relies on specified covariance models and parameters, which are complex and limited. In contrast, inverse distance weighting only considers the nearest points, lacking the advantages of global optimization and exhibiting stronger local effects.

Fractal geometry, with its ability to handle self-similarity and complex details, is particularly suited for modeling seafloor terrain, which often exhibits complex self-similar structures. Fractal interpolation functions can effectively capture the local features and variations in the data, making it well suited for handling noisy and complex data. This enables fractal interpolation to outperform traditional interpolation methods when dealing with noisy or structurally complex data.

Combining FIS with GPR creates a powerful model. FIS excels at accurately handling local features of the data, while GPR provides global regression, smoothing, and fitting. The combination of FIS and GPR integrates both local accuracy and global trends, enhancing overall modeling capability and avoiding biases that may arise from using a single method. This approach is suitable for various complex data modeling tasks, especially in scenarios involving irregularity, nonlinearity, noise, or missing data. Additionally, Perlin noise generates smooth and natural-feeling random fluctuations, offering the potential to optimize the details for 3D seafloor reconstruction, further enhancing the realism of the seafloor terrain.

## 5. Conclusions

This study addresses the limitations of traditional seafloor 3D reconstruction methods, which rely on dense measurement data, are costly, and struggle to capture complex terrain details. An innovative solution is proposed. The research is of significant importance for marine resource development, environmental monitoring, and seafloor disaster early warning, providing technical support for efficient reconstruction with sparse data. The study presents a hybrid modeling approach combining fractal algorithms and Gaussian process models. This method simulates the self-similar structure of the seafloor through an iterated function system for interpolation, enhancing the representation of local details, and uses Bayesian nonparametric regression to optimize global interpolation and quantify prediction uncertainty. The hybrid method significantly outperforms traditional methods in terms of accuracy, naturalness, and computational efficiency. The average error and mean square error were reduced by 86%, and the R^2^ goodness of fit improved by 80.7%, demonstrating its comprehensive advantage in capturing both local details and global trends. Additionally, leveraging the self-similarity principle of fractal interpolation, this method can extend to unmeasured areas, simulating points outside the measurement range and improving the reasonableness of terrain predictions for unmapped regions. Finally, after incorporating Perlin noise, the method excels in terrain naturalness and local detail restoration, compensating for the shortcomings of existing methods. Overall, the research provides a high-precision, high-efficiency technical solution for marine engineering and scientific research.

## Data Availability

The original contributions presented in this study are included in the article. Further inquiries can be directed to the corresponding author.
